# Toward Field Deployment: Tackling the Energy Challenge in Environmental Sensors

**DOI:** 10.3390/s25185618

**Published:** 2025-09-09

**Authors:** Valentin Daniel Paccoia, Francesco Bonacci, Giacomo Clementi, Francesco Cottone, Igor Neri, Maurizio Mattarelli

**Affiliations:** Department of Physics and Geology, University of Perugia, Via A. Pascoli, 06123 Perugia, Italy; francesco.bonacci@unipg.it (F.B.); giacomo.clementi@unipg.it (G.C.); francesco.cottone@unipg.it (F.C.); igor.neri@unipg.it (I.N.)

**Keywords:** optical sensors, RFID sensors, energy harvesting, distributed sensor networks, autonomous sensors

## Abstract

The need for sustainable and long-term environmental monitoring has driven the development of energy-autonomous sensors, which either operate passively or integrate energy harvesting (EH) solutions. In many applications, the energy cost of data transmission is a critical factor in autonomous sensing systems. To address this challenge, optical passive sensors, which exploit changes in reflectivity to monitor physical parameters, offer self-sustained operation without requiring an external power source. Similarly, RF-based passive sensors, both chipless and with minimal circuitry, enable wireless monitoring with low power consumption. When more energy is available, EH techniques can be combined with active optical sensors. Infrared laser-based CO_2_ sensors, as well as drone-mounted optical systems, demonstrate how EH can power precise environmental measurements. Beyond optics, other sensing modalities also benefit from EH, further expanding the range of self-powered environmental monitoring technologies. This review discusses the trade-offs between passive and EH-assisted sensing strategies, with a focus on optical implementations. The outlook highlights emerging solutions to enhance sensor autonomy while minimizing the energy cost of data transmission, paving the way for sustainable and scalable environmental monitoring.

## 1. Introduction

Monitoring the environment is a long-established practice, driven by the need to forecast adverse events in order to prevent them when possible or mitigate their effects. Compared to traditional methods based on personal observation and manual recording, the advent of distributed sensor networks (DSNs) has greatly increased the volume of available data, thus enhancing predictive capabilities [1]. However, this progress brings new challenges: increased complexity in data integration, which lies outside the scope of this review, and the urgent need for affordable sensors. Affordability must be understood not only as production and maintenance costs but also as environmental sustainability [2]. This demand, combined with the need to monitor a wide variety of signals, continues to drive the development of new types of sensors capable of integrating seamlessly with the environment and providing timely, actionable information.

A traditional, possibly exaggerated, example of a natural sensor is the rose bush in a vineyard [3]. By fortunate coincidence, the environmental requirements of roses (soil quality, irrigation, and weather) are quite aligned with those of grapevines. As such, monitoring the health of the rose bushes can offer early and accurate indications about the expected grape harvest. Even compared to artificial sensors, roses exhibit remarkable and desirable characteristics: complete energy autonomy (through soil nutrients and sunlight), continuous status indication (via flower quality), and passive long-range data transmission (through optical reflectance using ambient light). Thanks to their blooming period, they also offer early warning capabilities, allowing for timely countermeasures. These advantages, shared with other sentinel plants [4], are counterbalanced by significant limitations, such as the extreme specificity of the information provided and delayed responsiveness to stimuli.

In this review, we explore innovative sensing devices that seek to emulate key features exemplified by rose bushes, such as autonomy and biocompatibility. At the same time, these devices overcome their limitations by extending the range of measurable chemical and physical parameters and by achieving higher sensitivity. Our focus is particularly on the energy aspects of sensor design. This is a critical consideration in distributed sensor networks, where the cost of replacing batteries at each node can surpass that of manufacturing and installing the network itself. In practice, sensor nodes can be categorized based on the energy required for their operation. It is useful to distinguish between energy used for sensing and for data transmission. Based on these criteria, we classify sensors as active when the sensing process requires internal electronic power and passive when they rely on materials or components that respond to environmental stimuli by changing their physical properties without the need for a power supply. Similarly, active transmission systems require energy input, such as radio-frequency communication (e.g., LoRa). Passive transmission, by contrast, uses mechanisms like battery-free RFID tags or simple optical readouts [5,6]. For the purposes of this review, we consider only the energy expenditures at the sensor node itself, where energy is typically more limited, rather than at the receiving node, whether fixed or mobile.

The recent literature shows that, for common environmental parameters like temperature, humidity, and pressure, sensing energy remains very low, typically in the microwatt to low milliwatt range. Optical sensors and gas detectors may require more power but still generally consume less than the energy used in wireless data transmission [7,8,9]. Indeed, communication, especially over long-range protocols, is often the dominant energy expense in sensor node operation. These considerations are closely linked to affordability. Current developments in the field converge on two main directions: (i) materials and device architectures that minimize active driving or exploit ambient energy harvesting, thereby keeping sensing power modest and (ii) the use of printed or biodegradable platforms, along with battery-free communication strategies such as RFID or optical reflectance, which enhance both sustainability and cost-effectiveness [10,11,12,13].

This review aims to provide a comprehensive overview of such emerging approaches. It is organized as follows: for each class of sensors, we first present and discuss the underlying working principles, followed by selected examples of significant implementations relevant to environmental monitoring. We then compare the different sensor types and highlight perspectives for future deployment, with particular emphasis on their overall affordability.

## 2. Active Sensors

### 2.1. Autonomy Through Energy Harvesting and Power Management

The need for a power supply, whether for sensing or data transmission, does not preclude the possibility of energy-autonomous devices. By carefully managing power requirements, sensor nodes can operate by harvesting energy directly from the environment, in striking parallel to the energy strategy of rose bushes. In fact, the integration of active sensors with environmental energy harvesting is a growing field of research, especially for IoT applications related to Wireless Sensor Networks (WSNs). Typically, sensor nodes require batteries to provide a constant power supply, limiting their use in harsh environmental conditions, remote locations and operational lifetime. Several studies have shown that sensor nodes are becoming increasingly self-sufficient, relying on the energy harvested from the environment to operate without the need for an external power supply. This approach is particularly useful in terms of environmental sustainability, as it reduces the costs for deployment, and it enables strategies for long-term and maintenance-free sensor operation [6].

**Figure 1:**
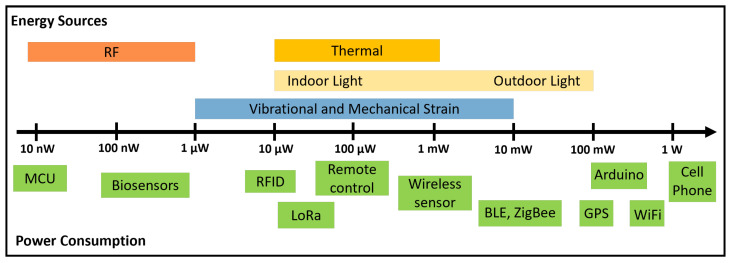
Comparison of power density ranges for various energy sources and power consumption ranges of common electronic devices. Colored horizontal bands indicate the typical or potential power densities (in W/cm^3^ or W/cm^2^) and power requirements of typical low-power modules. Data from [14,15,16].

Common energy harvesting techniques for Internet of Things (IoT) applications include the conversion of solar, thermal, kinetic, and electromagnetic and radio frequency (RF) energy into electrical power. The choice of the most suitable method depends on various factors, such as the specific use case, the availability and density of energy sources in the deployment environment, and the specific input power of the device. By enabling self-sustained operation, energy harvesting significantly reduces the need for battery replacements, hence lowering maintenance costs and minimizing the environmental impact linked to battery waste and conventional energy consumption. Figure 1 provides an overview of the main energy sources, highlighting their suitability for different IoT applications and typical power consumption.

In the specific case of environmental sensors, especially during constant activities like wireless data transmission, solar energy remains the most widely used and effective, particularly for outdoor deployments. Photovoltaic (PV) cells can generate power in the range of approximately 10–100 mW/cm^2^ under full sunlight [17], while indoor lighting conditions typically yield much lower power levels, around 10–100 μW/cm^2^, depending on the light intensity and the efficiency of the PV material. Despite being intermittently affected by factors such as shading, weather, and night cycles, solar energy remains attractive due to its high energy density and technological maturity. In [18], the authors developed a compact battery-free BLE ambient light sensor using a single photovoltaic (PV) cell to perform both energy harvesting and sensing, enabling low-cost, maintenance-free operation without external power sources, with a limit of detection of 200 lux.

Mechanical energy, particularly from ambient vibrations, is another valuable source and is commonly harvested using piezoelectric, electromagnetic, or electrostatic mechanisms. Piezoelectric materials can convert strain from structural vibrations, movement, or impacts into electrical energy, attaining power outputs between 10^2^ μW/cm^3^ and several mW/cm^3^ [16,19], depending on the material and vibration conditions (such as frequency and acceleration levels). Innovative piezoelectric materials are key enablers of efficient, miniaturized, and sustainable energy sources for self-powered IoT nodes. By converting mechanical energy into electrical energy, lead-free electroactive materials such as AlN [20], LiNbO_3_ [21], and PVDF [22] are able to power low-power electronics and support wireless transmission [23,24]. Lead-free flexible, thin-film, and nanoscale energy harvesters are also making it possible to integrate self-powered IoT nodes directly into structures, clothing, and human interfaces, which drastically reduces the need for battery replacements [19]. Electromagnetic and electrostatic harvesters operate under similar principles, with typical power outputs reaching up to several hundreds μW/cm^3^ in optimized environments such as industrial machinery or transportation systems [16].

Thermoelectric generators exploiting temperature gradients (Δ*T*) between different surfaces are used to harvest thermal energy. A few examples are the temperature difference between a buried sensor node and ambient air, between machinery and its surroundings, and even gradients existing on wearable devices; they can all generate continuous power output. Under intermediate temperature gradients, Δ*T* = 5–10 °C, typical for environmental or indoor applications, power densities around 100 μW/cm^3^ can be achieved [16]. In high-gradient industrial settings, this can increase to mW/cm^3^ scale, making it a reliable source for high-temperature-gradient conditions [25]. Thermoelectric systems for self-powered sensors have also been investigated, showing their potential to harvest energy from air to soil temperature differences and extend IoT node operational cycles [26].

It is also possible to harvest energy directly from radio frequency (RF) signals, such as Wi-Fi routers. In most real-world conditions, the power density of ambient RF energy rarely exceeds 1 μW/cm^2^. For instance, in environments with standard Wi-Fi signals at 2.4 GHz, the harvested power is often in the range of 0.01 to 0.1 μW/cm^2^ [27]. Similarly, cellular signals from GSM networks (900 or 1800 MHz) might offer power densities around 0.1 μW/cm^2^ to 1 μW/cm^2^ [27]. Despite its limited energy yield, RF energy harvesting can still be useful in specific contexts. It can support ultra-low-power applications, such as periodic wake-up functions in sensor nodes or the operation of passive devices like RFID tags and backscatter communication systems, which require minimal energy to function. In such cases, RF energy can support other harvesting methods or serve as a backup to maintain minimal sensor activity.

Power management is an important aspect of the sensor node as much as its energy harvesting capabilities. Efficient management strategies can enable the use of the sensor node in remote areas and in harsh environments and therefore a reliable deployment of a WSN. Active sensors generally require a continuous supply of energy to perform sensing, processing, and data transmission or reception. For this reason, an efficient power management unit must be designed in advance, taking into account the environmental energy sources available near the sensor and defining an appropriate energy budget for its operation. As discussed in [28], commercial microcontroller units (MCUs) such as PIC16LF, MSP430FR, and STM32L can typically use over 1 mW for data logging. Consequently, backup batteries are frequently necessary to provide supplemental power. However, in scenarios where battery deployment is not practical, such as in environments with limited space or harsh conditions, alternative strategies are required to guarantee a continuous energy supply for the sensor node [29].

**Figure 2:**
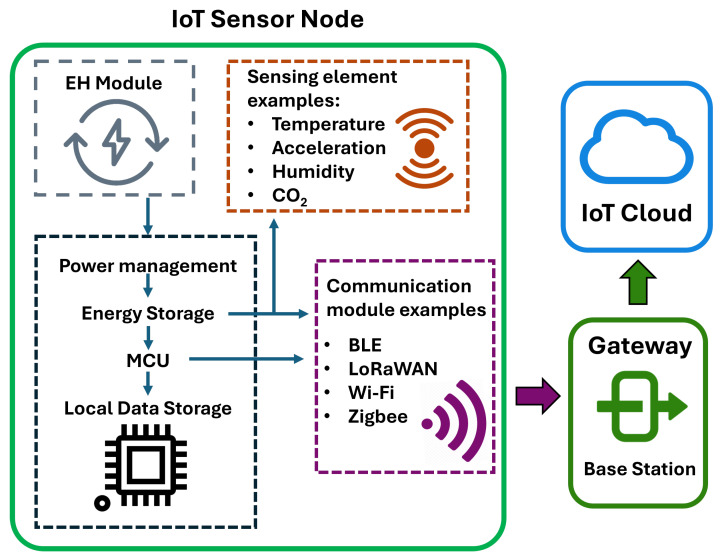
Example of node schematics: autonomous sensor, wireless communication with gateway and IoT cloud.

To ensure the reliable operation of energy harvesting sensor nodes, several strategies are commonly used to manage intermittent environmental energy sources as depicted in Figure 2. Excess energy is stored when available, allowing a node powered by an EH module to operate throughout unfavorable conditions using energy harvested during the day. Energy consumption is also minimized by only using power when necessary, such as avoiding data transmission if the sensed value has not changed. Additionally, systems can be optimized to reduce standby current and switch off non-essential components to prevent unnecessary energy loss. These principles can be applied to all forms of environmental energy harvesting, including solar, vibrational, thermal, RF, and hybrid solutions to ensure the limited and variable energy supply is used as efficiently as possible [30]. Thus, energy management circuits are essential for IoT nodes, often working in coordination with the MCU, which controls their operation and optimizes energy usage. They are designed to condition the low and irregular power from environmental sources so it can be used by a sensor or stored for later use. These circuits typically incorporate elements such as AC–DC conversion stages, voltage multipliers, and DC–DC converters to stabilize the output and supply the required voltage, and in some cases, they also employ nonlinear techniques to enhance energy extraction and improve conversion efficiency [31]. The regulated energy can be stored in elements like batteries or supercapacitors, or it can power the sensor directly. Modern energy management circuits may also incorporate maximum power point tracking (MPPT) algorithms, ultra-low-power supervisory units, and dynamic load adaptation to optimize efficiency [32]. This integration is crucial for enabling autonomous sensor systems to operate reliably under fluctuating environmental conditions while extending their lifetime and reducing the need for maintenance. Finally, the sensor node transmits its data using a suitable communication protocol, chosen based on the environment and required transmission range. This data is then sent to a gateway or base station and potentially uploaded to the IoT cloud, allowing users to access the information.

### 2.2. Triboelectric Nanogenerator (TENG)

In the field of environmental monitoring, triboelectric nanogenerators (TENGs) have emerged as an effective and promising technology for sustainable and self-powered sensing systems, especially in the context of energy harvesting by low-frequency mechanical excitations [33]. Their high sensitivity to random mechanical stimuli and lightweight structure make them ideal devices for real-time monitoring of environmental parameters such as air and water quality [34] and mechanical vibrations [35].

#### 2.2.1. Working Principle

**Figure 3:**
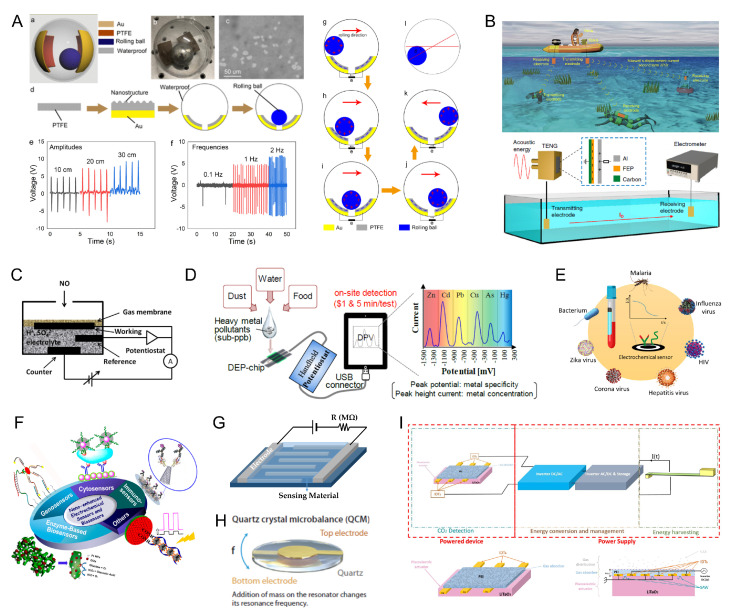
Non-optical sensors for environmental monitoring. (**A**) Example of a freestanding nanogenerator based on a rolling contact mode, showing: (a) device design, (b) photograph of the device, (c) SEM image of the PTFE rough surface, (d) fabrication process, (e, f) output voltages demonstrating a dependence on wave amplitude and frequency, enabling real-time characterization of the local wave spectrum, (g–l) working principle. Adapted with permission from [42] under CC BY License. (**B**) Schematic diagram of wireless underwater communication using TENG-generated Maxwell displacement currents. Adapted with permission from [55] under CC BY License. (**C**) Schematic of an amperometric electrochemical gas sensors using the three-electrode scheme. Reproduced with permission from [58] under CC BY License. (**D**) Work flow of the “DEP-On-Go” sensor [59] for the detection of several heavy metal ions in environmental samples. Reproduced with permission from [59] under CC BY License. (**E**) Electrochemical (bio)sensors can be used for the diagnosis of viral and tropical diseases. Reproduced with permission from [60] under CC BY License. (**F**) Sketch of the various functionalization strategies of electrochemical sensors for highly selective analyte sensing. Reproduced with permission from [61] under CC BY License. (**G**) Schematic view of a chemiresistor sensor based on analyte-induced resistance changes. Reproduced with permission from [62] under CC BY License. (**H**) Schematic draw of a quartz-crystal microbalance sensors. Reproduced with permission from [63] under CC BY License. (**I**) Autonomous sensor based on surface acoustic wave (SAW) frequency shift for carbon dioxide detection. Adapted with permission from [64] under CC BY License.

Triboelectric nanogenerators harvest mechanical energy by coupling the triboelectric effect, where two materials become oppositely charged upon contact, with electrostatic induction, which drives a current through an external circuit when the charged surfaces move relative to one another [36]. The possibility of using various operational schemes [37] allows TENGs to be customized to a wide variety of ambient energy scenarios, from low-frequency human motions [38] to high-amplitude environmental vibrations [33]. Typical architectures include tandem-disk (TD-TENG) [39], torus-structured (TS-TENG) [40], and rolling-ball (RF-TENG) [41,42] nanogenerators. When activated by low-frequency vibrations (0.1–2 Hz), they typically provide power densities on the order of several μW/cm^2^, average open-circuit voltages of several V, typical short-circuit currents in the μA range, and max peak powers of a few tens of μW. As an example, Figure 3A showcases a typical rolling-ball design [42], comprising a stainless ball that is free to roll within a spherical waterproof shell, periodically contacting stationary triboelectric layers made of nanostructured PTFE. The rolling motion induces a reversed potential difference between the gold electrodes, generating alternating current. Since conventional TENGs output high voltages with relatively high frequencies or in the form of AC pulses, dedicated power management modules (typically including a rectifier and, in some cases, a voltage transformer such as a DC buck converter module [37]) are generally required to power sensors or charge energy storage components.

#### 2.2.2. Applications

TENGs are extensively used for real-time water quality monitoring [34], particularly in marine environments that are increasingly exposed to pollution and degradation from human activities. Combined with electrochemical sensors, biosensors, and integrated with MEMS-based chips, they can be used to measure many different water parameters such as pH [43], temperature [40], and concentration of organic and inorganic contaminants [39]. TENGs are not only used to simply power off-the-shelf distributed sensors but have also been engineered as active sensing elements. An example is the rolling-ball TENG [42] in Figure 3A, which produces voltage outputs correlated with the ocean wave frequency and amplitude, enabling real-time characterization of the local wave spectrum. Using a 1.5 cm stainless steel solid sphere, the authors reported output voltages of 4.8 V, 7.1 V, and 8.5 V when the device was subjected to 1 Hz water waves with amplitudes of 10, 20, and 30 cm, respectively. Other device architectures have been employed for impulsive force sensing [35], detection of Hg^2+^ ions [44], and real-time monitoring of water levels [37].

Because most conventional devices are expensive and non-biodegradable, leading to increased production of e-waste, recent years have witnessed considerable efforts toward developing so-called green TENGs [13], i.e., generators based on eco-friendly and biocompatible materials, with significant implications for sustainable environmental monitoring. Various approaches have been explored to replace conventional triboelectric polymeric layers with environmentally friendly alternatives, such as food scraps [45], animal- and plant-based materials [46], and recyclable and non-recyclable industrial wastes and other daily-use materials [47]. These devices typically show comparable energy performance to those based on synthetic materials, with open-circuit voltages of the order of hundreds of V, short-circuit current density of few μA/cm^2^, and power density of hundreds of μW/cm^2^.

Moreover, since charge generation in the triboelectric layer is influenced by water vapor, variations in the electrical output of TENGs can be directly correlated with humidity levels, enabling their use as self-powered humidity sensors. This sensitivity is further amplified when using biomaterials due to their inherently high hydrophilicity or hygroscopicity. Luo et al. [48] developed a plant leaf-based triboelectric nanogenerator (LPL-TENG) for smart agriculture, which allows the monitoring of plant leaf humidity with a sensitivity of about −3.0 V/% RH. Zhang et al. [49] used half-cell allium plant skins (e.g., leek skin) as triboelectric material to detect moisture variation in the range of 25–56%. We also mention the biocompatible polydopamine-based TENG reported by Panda et al. [50] (sensitivity of 1.55 V/10 % RH over the range 25–92%), and the white sugar-based TENG designed by Liu et al. [51]. Other notable examples for environmental applications include the wind speed sensor device fabricated from discarded peanut shells [52], the self-powered arc nanogenerator (AS-TENG) made from milk cartons for real-time water quality (pH) monitoring by Zhou et al. [53], and the agricultural sensing system based on corn husk composite films developed by Gu and co-workers [54], which enables simultaneous collection and wireless transmission of farmland temperature, humidity, light intensity, and soil moisture.

Finally, TENGs can also be used for wireless underwater communication, as they can generate low-frequency Maxwell displacement currents that can propagate through water and are not critically disturbed by salinity, turbidity, and underwater obstacles [55], Figure 3B. See also ref. [56,57] for recent developments of TENGs in the field of underwater wireless communication.

TENG-based energy harvesters and self-powered sensors, including both conventional and sustainable designs, have been extensively validated under laboratory conditions. However, large-scale deployment remains challenging due to stability, flexibility, and durability issues under harsh conditions, highlighting the need for improved power management and material durability to ensure long-term reliability.

### 2.3. Electrochemical Sensors

The acceleration of the world’s industrialization is resulting in extensive release of hazardous chemicals into the environment [65], which poses serious risks not only to environmental health but also to human safety. In this context, electrochemical sensors and biosensors have gained considerable attention due to their ability to offer rapid, on-site detection of both biological and chemical contaminants with high specificity and sensitivity [66].

#### 2.3.1. Working Principle

Electrochemical sensors detect analytes by converting chemical interactions (usually a redox reaction) at the electrode surface into measurable electrical signals, proportional to target analyte concentration. In biosensors, the sensing electrode is usually functionalized with biological recognition elements such as DNA, enzymes, antibodies or aptamers that specifically interact with the target analyte. Electrochemical (bio)sensors typically operate using various techniques, including amperometric, voltmetric, and impedimetric methods, with the three-electrode scheme being the most widely adopted one. Figure 3C presents an example of a three-electrode amperometric sensor used to detect ambient nitric oxide [58]. It consists of a working, a reference, and a counter electrode, immersed in an electrolyte and enclosed by a gas-permeable, liquid-impermeable membrane. When the target gas diffuses through the membrane and undergoes a redox reaction at the working electrode, a current flows between the working and counter electrodes, with the reference electrode maintaining a constant potential to ensure accurate and stable signal output. In voltmetric sensors, the current is measured as a function of varying potential, generating current-voltage curves for analysis. Instead, impedimetric sensors operate by measuring changes in electrical impedance at the electrode–electrolyte interface, which occur as a result of the interaction between the target analyte and a recognition layer.

#### 2.3.2. Applications

Electrochemical sensors are widely used for monitoring environmentally hazardous gaseous pollutants [58,67]. They offer reliable and real-time detection down to the parts-per-million (ppm) level. Another important utilization is for the sensitive detection of pesticides in groundwater and soil [66], as well as heavy metal ions in environmental and food samples [59,65,68] (Figure 3D). In this context, a particularly promising application is the detection of phenolic compounds such as catechol and hydroquinone [69,70], which are major environmental pollutants due to their extensive use in various industrial processes. Electrochemical biosensors have also been used in the context of healthcare for the detection of pathogens and viruses [60,71] (Figure 3E).

In recent years, the integration of advanced nanomaterials has significantly enhanced the performance of these sensors [61]. Nanomaterials such as graphene and carbon nanotubes [72,73] and polymer nanocomposite [74], metal, and metal–organic nanoparticles [75] offer unique advantages, including high surface area, enhanced electron transfer properties, and the possibility to be functionalized with specifically designed biorecognition tags, leading to highly sensitive biosensing (Figure 3F).

Despite these recent advancements, most electrochemical sensors still rely on substantial external energy sources to power potentiostats, galvanostats, or signal transducers, limiting their use in environmental monitoring, where power access and maintenance are often constrained. A promising alternative is represented by self-powered electrochemical sensors (SPESs), which detect analyte concentrations without the need for an external power supply by exploiting the electricity self-generated through spontaneous reactions occurring in a galvanic cell [76]. SPESs are primarily based on biofuel cells, which operate without costly metal catalysts by using oxidoreductases, enzymes, organelles, or microorganisms as biocatalysts [77]. Other SPES types include photoelectrochemical (PEC) devices, where reactions are triggered by light absorption [78], as well as systems based on liquid-activated batteries and ion-selective electrodes [76]. Their inherently simple two-electrode architecture supports compact, miniaturized designs, ideal for field-deployable environmental sensors. Emerging applications include SPESs for detecting heavy metals in polluted water [79], tracking pH changes in river samples [80], and monitoring the degradation of real oilfield wastewater [81].

However, while SPESs significantly reduce the need for external excitation sources, their practical implementation is still constrained by limited energy generation. Due to small sample volumes and restricted electrochemical kinetics, they typically produce low open-circuit voltages (often <1 V) and output currents in the microampere range, which limits their ability to power additional electronic components. In environmental monitoring, where fully autonomous operation is highly desirable, innovative strategies for energy management and system integration are therefore essential.

### 2.4. Active Optical Sensors

Environmental monitoring often relies on optical sensing techniques due to their versatility, sensitivity, and the non-contact nature of measurements. Optical sensors exploit the interaction between light and the environment to extract information about specific physical or chemical parameters. Unlike passive optical sensors, active optical sensors include their own powered light source instead of relying on ambient illumination. For this reason, their energy budget must account not only for the sensing and data transmission components but also for powering the light source. Measuring the light passing through a medium, such as air, water, or a suspended particulate and the amount of light absorbed at specific wavelengths by the molecules, it is possible to reconstruct which molecules have interacted with the light. This is the fundamental principle behind non-dispersive infrared (NDIR) and tunable diode laser absorption spectroscopy (TDLAS) sensors. Using the Beer–Lambert law, it is also possible to estimate the concentration of the substance from the light intensity. This principle is, for instance, widely used for monitoring gases like CO_2_, CH_4_, and NO_X_ [82,83,84,85]. Similarly, analyzing the reflected light, it is possible to extract information about the environment. This approach is used to quantify the health and density of vegetation through vegetation indices [86,87] and to estimate pollutant deposition [88,89]. Measuring the deflection of light (scattering) as it encounters particles or inhomogeneities in the medium, it is possible to estimate particulate matter (PM) concentrations [90,91]. A particular kind of scattering technique is Raman scattering, used to detect specific molecules or pollutants [92]. This method, however, involves the use of a particular light source (typically a laser) and is based on measuring inelastically scattered photons at different wavelengths. Although powerful, this technique often requires higher energy and more complex optics, making them less common in energy-constrained field applications.

Several studies have demonstrated self-powered or ultra-low-power CO_2_ sensing systems suitable for autonomous environmental deployments. Liu et al. [93] described a battery-powered portable NDIR-based CO_2_ sensor optimized for atmospheric mapping of UAVs, achieving a precision of ±1 ppm at 1 Hz. Although not fully powered by energy harvesting, its low-power architecture supports extended autonomous operation.

Tinivelli et al. [94] presented a low-cost, energy-autonomous, wireless sensing system for environmental sensing, designed for continuous in situ monitoring of dissolved carbon dioxide (dCO_2_) in natural freshwater environments [94]. The system integrates a sub-3 mW infrared sensor, capable of measuring dCO_2_ alongside complementary environmental parameters such as salinity, conductivity, and temperature. This combination of multi-parameter sensing is critical for understanding complex geochemical processes in groundwater, lakes, and rivers, especially in regions prone to seismic or volcanic activity where CO_2_ fluxes can act as early warning indicators. The sensor node minimizes power consumption and is designed to operate for extended periods, coupling a buffer battery with ambient energy sources (e.g., photovoltaic harvesting or river turbine). The reported energy consumption for a cycle of acquisition, storage and transmission is about 10 mWh [94].

Recent advances in environmental sensing have demonstrated the viability of autonomous optical sensors based on light scattering, enabling continuous monitoring of particulate and gaseous pollutants in the field [95,96,97]. On the inelastic side, Raman LiDAR has emerged as a powerful tool in environmental monitoring. A recent system successfully demonstrated remote detection of CO_2_ leakage over carbon capture sites using Raman scattering [98], with an accuracy greater than 99.8% compared to in situ instruments. Although current field versions remain power-heavy, with further energy optimization and management, it could become fully self-powered.

Autonomous active optical sensors, such as laser-based spectrometers, appear only as proofs of concept or prototypes and remain scarce as commercial solutions despite their clear potential for environmental monitoring and autonomous platforms. The primary barrier lies in the substantial energy expenditure required to drive their light sources, typically high-power lasers or LEDs, which far exceed the capabilities of current low-power electronics and energy harvesting modules. Unlike passive optical systems, which can rely on ambient light, active systems must generate and emit their own illumination, leading to high instantaneous power draws that conflict with the limited energy budgets of battery-powered or energy autonomous devices.

### 2.5. Other Non-Optical Sensors

Other examples of non-optical environmental sensors include capacitive devices, which detect changes in capacitance in the presence of an analyte [99], and chemiresistive sensors, where gas exposure alters the material’s electrical resistance through chemical interaction (Figure 3G). Other sensors exploit changes in acoustic resonance frequency due to surface mass variations from gas adsorption, such as quartz-crystal microbalance (QCM) and surface acoustic wave (SAW) sensors [63]. QCM sensors detect analyte-induced mass changes by measuring shifts in the resonant frequency of a quartz crystal coated with a sensing material, which deforms under an applied alternating electric field to generate standing acoustic waves (Figure 3H). An example of an active SAW-based gas sensor integrating a piezoelectric energy harvester with a SAW sensing unit to enable autonomous CO_2_ detection can be found in ref. [64] (Figure 3I). A flexible piezoelectric beam converts ambient mechanical vibrations into electrical energy to power the sensor. The SAW sensor is based on a PEI (polyethylenimine) sensing layer that selectively absorbs CO_2_. This alters the surface mass and stiffness, causing the resonant frequency to shift proportionally to the gas concentration. The shift is measured using frequency detection circuits and processed by low-power microcontrollers, enabling autonomous detection down to 10 ppm.

## 3. Passive Sensors

### 3.1. Passive Optical Sensors

The working mechanism of roses as sensors, i.e., the change in optical response under an external stimulus, is shared by many passive environmental sensors. These devices combine the advantage of remote interrogation, requiring no internal power source, with enhanced specificity achieved through engineered designs, enabling sensitivity to a wide range of chemical and physical parameters. In passive optical sensors, a change in temperature, humidity, pressure, pH, or a chemical analyte alters the material’s optical properties, which can be read by the eye or an optical detector (far from the sensor node). Unlike active sensors, where the light source is usually internal to the sensor node with a relatively significant power consumption, passive optical sensors exploit ambient light and primarily rely on changes in reflectance. This effect can be obtained by two different mechanisms: a change in the optical interference due to a structural modification of the sensing element, as in photonic crystal based sensors [100] or a change in absorption properties of the material, due to a molecular or phase change, as a consequence of the change in the measured parameter [101,102,103].

In particular, in photonic crystals (PhCs), the color is the result of interference between the light reflected at the multiple interfaces of a spatially ordered structure (1D, 2D, or 3D), with lattice parameters comparable to the wavelength of light. Therefore, any change affecting the refractive index, such as adsorption or immobilization of chemical species or structural modifications causing changes in the interplane distance, induces a measurable shift of the reflected wavelength. These properties make the system an effective sensor of several physical and chemical parameters [104,105].

Molecular chromogenic materials and plasmonic sensors both rely on changes in the absorption spectrum to produce a visible color shift. In chromogenic materials, engineered molecules undergo molecular or phase transitions in response to physical stimuli (such as temperature or light) or chemical ones (such as gases, water, or pH), resulting in a change in the absorption spectrum. In plasmonic sensors, the strong localized surface plasmon resonances of metal nanoparticles shift due to analyte binding or changes in the surrounding refractive index [106].

#### Applications

**Figure 4:**
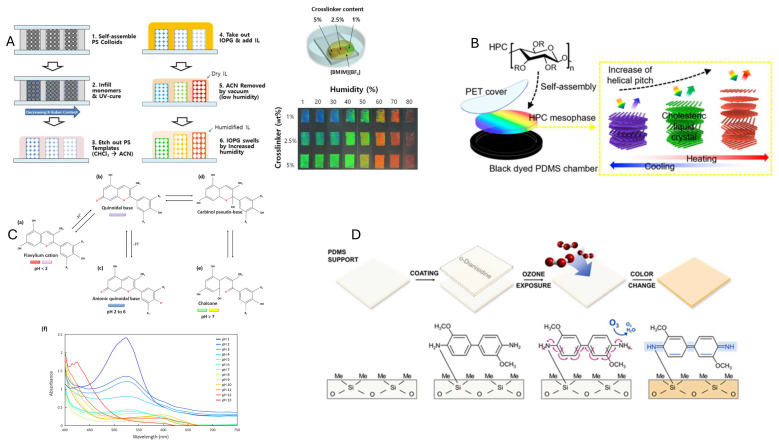
Passive optical sensors for environmental monitoring. (**A**) Example of a colorimetric humidity sensor using inverse opal photonic crystal, showing the device design and working principle, and the visual experimental results. The color of the film depends on the swelling related to humidity. Adapted from [108] under CC BY License. (**B**) Example of a colorimetric temperature sensor based on cholesteric liquid-crystal structure. Schematic image showing the materials and the sensing principle. The system is fabricated using hydroxypropyl cellulose (HPC) and ethylene glycol. The temperature increase causes a change in the helicoidal pitch of the liquid crystal. Adapted from [109] under CC BY License. (**C**) Example of chromogenic sensor based on molecular changes. The anthocyanin in red cabbage undergoes structural transformations across different pH ranges from (a) flavylium cation (pH: <2) to (b) quinoidal base, (c) anionic quinoidal base, and (d) carbinol pseudo-base (pH: 2–6), and (e) chalcone (pH: >7). These changes induce significant changes in the UV-Vis absorption spectra (f). Reproduced from [113] under CC BY License. (**D**) Schematics of the preparation and working principle of o-dianisidine-based ozone passive sampler. The sensible material is coated on a PDMS support; the presence of ozone induces a change in chemical bonds in turn provoking a change in the color of the material. Reproduced from [114] under CC BY License.

Fiber Bragg gratings and waveguides, although widely used as photonic crystal-based optical sensors, require light to be coupled into the fiber. This limits their compatibility with ambient illumination conditions [107]. In contrast, passive sensors typically rely on multilayer structures deposited on a substrate to form 1D PhCs [108,109,110] or on self-assembled sub-micron particles forming opal-like 3D PhCs [111,112]. In this category, temperature and humidity sensors are the most common. Recently, using multilayers of vinyl polymers with different refractive indexes, Jung et al. [108] demonstrated a chemically tunable device able to measure humidity in a 10 to 90% RH range, with a response in the order of seconds (Figure 4A). A similar material shows simultaneous responsivity to both temperature and humidity, although reducing specificity [110]. A layered structure is also present in cholesteric liquid crystalline, where the pitch of the characteristic helicoidal structure can depend on external parameters. Using the biodegradable polymer hydroxypropyl cellulose, Yi et al fabricated a stable device operating between −20 and 25 °C, a temperature range suitable for environmental monitoring [109] (Figure 4B).

While chromogenic and plasmonic sensors can be used for measuring physical parameters, exploiting structural mechanisms similar to PhCs [115], their most important application is for chemical sensing, in particular of pollutants [116]. As an example of a sustainable chromogenic sensor, a thin film composed of biomass-derived chitosan impregnated with natural red-cabbage anthocyanin dye has been devised as a pH indicator with good sensitivity (seven steps in the pH scale) [113]. The sensor was shown to maintain its stability and color fidelity even after exposure to an aqueous environment, making it potentially useful for on-site water or soil pH monitoring (Figure 4C). Choi et al. [114] demonstrated a colorimetric ozone badge made by impregnating the redox dye o-dianisidine into a polydimethylsiloxane sheet. Ozone presence oxidizes the dye, turning it yellow with a progression dependent on concentration, with high sensitivity (few ppb), also in the presence of interfering gases (Figure 4D). Plasmonic sensors are often exploited for detecting trace concentrations in laboratory conditions, but if the nanoparticles are embedded in a film, the system can also be used for real-time monitoring. For instance, Zhai et al. [117] showed a realization of an H_2_S sensor using Ag nanoparticles in gellan gum. In the presence of the chemicals, the nano metallic Ag strongly binds H_2_S, forming Ag_2_S; this reaction converts the yellow AgNP film to colorless, yielding a clear visual change, which is both highly sensitive (<1 μM) and linear in a wide range (up to 85 μM).

Natural organisms, such as plants, are environmental sensors, although with low specificity, and this has led to the development of artificial inorganic engineered sensors. However, recent advances in biotechnology have allowed the realization of new living colorimetric sensors. In a brilliant combination of different technologies, Chemla et al. [118] demonstrated the use of bacteria with a bioengineered reflectance fingerprint for monitoring the environment. Making the bacteria detectable by the remote observation of hyperspectral cameras (shown up to 90 m), they can be used as in-field sensors, giving indications on soil conditions.

### 3.2. Passive RF Sensors

Passive radio frequency (RF) sensors derive the necessary energy for sensing and communicating directly from the interrogating signals emitted by a reader [119]. This powering method eliminates the need for onboard energy storage, making them suitable for long-term, low-maintenance deployments, especially in remote or inaccessible locations. This section will explore the most promising passive RF technologies, examining their operational principles and highlighting notable applications from the recent literature. We will cover inductor–capacitor (LC) resonant sensors, radio frequency identification (RFID) based sensors, and surface acoustic wave (SAW) sensors.

#### 3.2.1. LC and RFID

**Figure 5:**
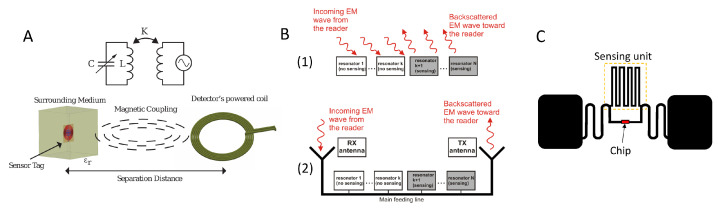
Passive RF sensors for environmental monitoring. (**A**) Example of an LC resonant sensor for moisture sensing. Reproduced with permission from [120] under CC BY License. (**B**) Schematic of two different architectures of chipless RFID sensors based on reflection (1) or retransmission (2). Reproduced with permission from [121] under CC BY License. (**C**) Example of a passive electromagnetic RFID tag. Reproduced with permission from [122] under CC BY License.

LC and RFID sensors represent a broad category of passive devices that operate on the principle of electromagnetic resonance, combined with either inductive coupling or backscatter for communication. While their implementations vary from simple two-component LC circuits to complex chip-based RFID tags, many of them share fundamental mechanisms for power transfer, communication, and sensing, leading to significant overlap in their applications (Figure 5).

##### Working Principle

An LC sensor is a simple resonant circuit whose resonant frequency $\left(f_{0}=1/\left(2\pi \sqrt{LC}\right)\right)$ is determined by its inductance (L) and capacitance (C) [123] (in practice, the parasitic resistance of the inductor makes it an RLC circuit [124]). The sensing function is achieved by designing the capacitor (or, less commonly, the inductor or the resistor) to be sensitive to a specific environmental parameter. A reader then interrogates the sensor with a coil via near-field inductive coupling over very short distances, typically a few centimeters. As the environmental parameter fluctuates, the reader detects changes in the resonant frequency, input impedance, or Q-factor, thereby measuring the parameter [123,124].

RFID technology expands on this principle and is also able to operate in the far-field using backscatter as the communication method [125]. The sensor tag receives an RF signal from a reader, modulates it, encoding its ID and sensor data, and reflects or retransmits (or backscatters) a modified version back to the reader [121,122,126]. The effective range of these systems is a major challenge, as the power of the signal returning to the reader ($P_{r}$) is governed by the radar equation, which shows a dramatic falloff with distance (*r*) [127].

(1) $$P_{r}=\frac{P_{t}G^{2}\lambda^{2}\sigma }{{\left(4\pi \right)}^{3}r^{4}}$$

The relationship between received power and distance $P_{r}\propto r^{-4}$ indicates severe signal loss with distance, which significantly limits read ranges, especially in real environments where signal leakage, coupling, and reflections from surrounding objects have to be considered [128].

There are two main classes of passive RFID sensors, distinguished by the absence or presence of an integrated circuit (IC).

- Chipless RFID: the physical structure of the tag acts as both the sensor and the identifier. The tag is generally designed with multiple resonators to have a unique spectral signature (a specific pattern of resonances across different frequencies) and, like LC sensors, some of these resonators can be engineered to be sensitive to an environmental parameter. Sensor data is therefore analogically encoded into the reflected signal.
- Chip-based passive RFID: these tags incorporate an IC and use RF harvesting: a small fraction of the energy from the reader’s RF signal is converted into DC power by a rectifier circuit to operate the tag’s components [129]. These tags can be divided into the following [122]:
  − Electromagnetic: the antenna itself is the sensor, similarly to a chipless or an LC sensor.
  − Electronic: the sensing and communication functions are separate. The tag consists of an antenna, an IC and a sensor, and sensor data is therefore digitally encoded in the backscattered signal.

A key limitation for a chipless tag is its linearity, which makes the tag’s already weak signal spectrally indistinguishable from reflections from surrounding objects, significantly limiting its read range [128]. Chip-based tags overcome this because the IC’s active modulation of the RF signal is a nonlinear process, creating frequency components that are spectrally distinct from linear environmental noise. To increase the read range, one could break the LTI property [130]. There are two ways to accomplish this.

- Break linearity by introducing a nonlinear element, such as a diode. The tag then becomes a harmonic transponder [130], a device that backscatters the signal at a harmonic frequency [131].
- Break time-invariance, for example, by using the micro-Doppler effect [130,132].

Another potential improvement to read range is using cross-polar tags. Changing the polarization of the backscattered signal should naturally isolate the tag’s response from background clutter. However, this generally does not lead to an increase in read range as this method’s effectiveness heavily depends on the entire implementation of the RFID system [133].

An extension of far-field harvesting is the emerging paradigm of Ambient Backscatter Communication (AmBC), i.e., harvesting energy from and communicating by modulating pre-existing ambient RF signals, thus removing the need for a dedicated reader to provide power [134,135]. However, AmBC faces significant technical challenges, primarily the detection of extremely weak backscattered signals amidst strong ambient noise and interference [136].

##### Applications

The versatility of LC and RFID sensors has led to their application in a wide range of environmental monitoring scenarios. The choice of technology often depends on the required read range, cost, and the specific parameter being measured.

A primary application for these sensors is monitoring moisture, which relies on detecting changes in the dielectric permittivity of the surrounding environment [120]. In precision agriculture, LC sensors have been developed with optimized capacitor geometries for higher sensitivity to moisture, with interdigital capacitor (IDC) designs exhibiting 10% increased sensitivity in frequency offset and a 5% increase in detection distance compared to parallel plate capacitor (PPC) designs [120]. Other LC sensors have been developed with inductively coupled extenders to increase read range for deep soil monitoring, achieving a sensitivity of 4.52% moisture content/MHz and extending read range to over a meter into the soil [137]. Chipless RFID sensors have also been created for this purpose, using cross-polarization to measure subsoil moisture, demonstrating a wireless reading distance of 50 cm (at 9–10 cm depth) and sensitivities between −3.92 MHz/VWC and −4.85 MHz/VWC [138]. Similar sensors have also been made from biodegradable materials to improve sustainability and reduce maintenance and recovery costs, such as paper-based devices that measure leaf wetness [139], PLA-based devices that measure soil moisture and show a minimum sensitivity of ∼9 MHz/VWC [140], and soil pH sensors which measure the dissolution of SRRs made of octacalcium phosphate-coated magnesium compared to reference SRRs [141]. Concurrently, chip-based RFID technologies offer more complex solutions. Some simply modify regular RFID tags, using the strength of the tag’s signal to estimate soil moisture, able to detect 20 mL of moisture from a distance of 0.8 m [142]. More advanced designs put sensors on a tag using a smartphone with NFC as a reader [143]. For large-scale deployment, comprehensive systems have been designed that combine passive RFID sensors with mobile data collection vehicles and long-range LoRa communication, creating a scalable monitoring network [144]. The same concept is applied in civil engineering, where passive sensors are embedded to monitor the internal humidity of concrete for structural health assessment [145,146].

Biodegradable sensors have also been developed for medical monitoring to avoid secondary surgeries to remove medical implants. For example, a bioresorbable wireless pressure sensor has been developed using Mg, Zn, and PLGA, which operated for up to 4 days in a rat model for intracranial pressure monitoring [147]. Similarly, another biodegradable LC pressure sensor was microfabricated from zinc and PLA for short-term biomedical applications, remaining stable and functional for 86 h [148]. For orthopedic applications, a pressure sensor fabricated with Mg conductors on a polymer substrate was observed to maintain its functionality for 10 days in aqueous conditions [149]. A comprehensive review describes the use of various biodegradable and renewable materials, including biopolymers, natural fibers, and agricultural byproducts, for microwave antennas. These materials enable the development of green IoT sensors for a wide range of applications, including agriculture, environmental and civil infrastructure monitoring, healthcare, wearable electronics, food and beverage, and logistics [12].

More recently, researchers demonstrated a self-powered temperature sensor that harnesses the membrane potential of living cells as an energy source, highlighting a promising direction for bio-integrated and sustainable sensing technologies [150].

Another important application in structural health monitoring is strain and crack detection. The core principle is that mechanical strain or the formation of a crack physically changes the sensor’s properties. Highly sensitive LC sensors have been developed for measuring strain, with one design showing a sensitivity of 121.9 kHz/0.01 ϵ [151], but also dual-resonance designs capable of measuring both temperature and strain simultaneously have been studied, achieving a temperature sensitivity of 27.3 kHz/°C and a strain sensitivity of 100 Hz/μϵ [152]. Using chipless RFID technology, high sensitivity to strain (36.32 kHz/μϵ) has been achieved by using advanced materials like laser-induced graphene [153] or by engineering the sensor with nanocracks that significantly alter its signal when stretched, demonstrating a gauge factor of 50 for strains under 1% [154]. Chipless RFID sensors for detecting cracks have also been developed, with one design showing a frequency shift of 15.5% for a 0.2 mm wide, 2 mm deep, 4 mm long crack [155], including smart antennas that can determine a crack’s direction and size based on how their signal changes [156]. Similarly, chip-based RFID technology has been effectively used for both strain and crack detection. Several designs use the deformation of a patch antenna to measure strain [157,158], with one, for example, being a strain–crack sensor which achieves a sensitivity of 0.81 kHz/μϵ at 910 MHz of resonant frequency [159]. For crack sensing, approaches include measuring the signal shift as a crack alters the antenna’s properties, reaching a sensitivity of 3.94 MHz/mm of crack depth [159], detecting a sudden signal failure when the antenna breaks due to the crack [160], using dual-tag systems for enhanced monitoring [161], and applying machine learning for automatic crack characterization [162].

Another important application for passive sensors is gas sensing, which typically relies on a sensitive material that changes its electromagnetic properties upon adsorbing a target gas. LC resonant sensors have been widely developed for this purpose, though many require elevated temperatures for optimal performance. One such sensor utilized 2D Tin Disulfide (SnS2) on a robust LTCC substrate to detect NO2, achieving a detection limit of 0.6 ppm [163], but required heating to 120 °C. Similarly, ZnO nanowires were also employed to sense NO2 in a novel structure which minimized substrate interference, demonstrating a detection limit of 0.5 ppm [164], with an optimal operating temperature of 180 °C. For ammonia (NH3) detection, an LC sensor was developed using a PANI/CNT composite [165] that performed best at 45 °C with a sensitivity of about 0.04 MHz/ppm. In contrast, other designs work at ambient temperature. For instance, inkjet printing was used to create a chipless RFID sensor with a carbon nanotube-based ink for CO2 detection using a dual-resonator design where one resonator works as a stable reference [166], demonstrating 1 dB of RCS shift going from low to high CO2 concentration. In the context of smart agriculture, chipless RFID sensors have been developed for the detection of gases like CO2, NH3, and ethylene [167]. Chip-based RFID sensors offer more functionalities. For example, “the principle of self-correction of individual multivariable vapor sensors against temperature fluctuations” was demonstrated for water vapor [168]. More complex systems integrate an entire commercial methane sensor into a passive RFID tag [169].

Passive LC and chipless RFID sensors built on ceramic substrates offer a robust solution for high-temperature monitoring because they do not contain semiconductor components. For instance, an LC pressure sensor based on LTCC has shown stable operation up to 400 °C [170] and a temperature sensor on the same material up to 700 °C [171], while a design using an HTCC substrate with platinum conductors has enabled combined pressure and temperature sensing at up to 1200 °C [172]. Similarly, a chipless RFID temperature sensor has been demonstrated up to 370 °C [173] and a tag based on an alumina substrate and platinum conductor has been proposed for temperature measurements up to 1000 °C [174]. Other types of harsh environments have also been explored [175]. For example, flexible, chipless RFID sensors have been developed for pipeline integrity monitoring to detect coating failure due to corrosion [176].

#### 3.2.2. Surface Acoustic Wave Sensors

Surface acoustic wave (SAW) sensors are a versatile platform for passive, wireless sensing. SAW devices use surface acoustic waves on piezoelectric materials to detect a wide range of physical, chemical, and biological parameters, making them ideal for long-term environmental monitoring [177].

**Figure 6:**
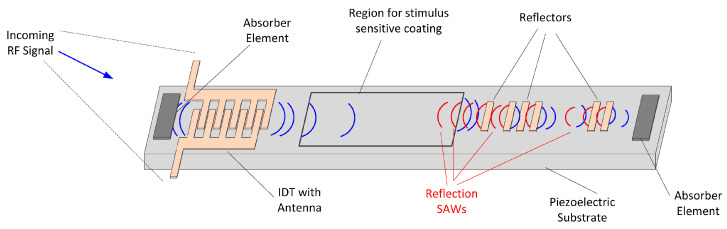
Surface acoustic wave (SAW) sensor with delay line architecture and ID capabilities. Reproduced with permission from [178] under CC BY License.

While SAW sensors use backscatter as a communication method, the surface acoustic wave introduces a characteristic time delay in the retransmitted signal, as shown in Figure 6. This delay allows SAW tags to be read with much lower power and in more challenging, noisy environments compared to other passive RF technologies [178].

##### Working Principle

A SAW sensor’s operation relies on the piezoelectric effect. On a piezoelectric substrate, an Interdigital Transducer (IDT) converts an incoming RF signal into a mechanical surface acoustic wave. This wave travels along the substrate, where its properties can be altered by external physical, chemical, or biological parameters. Finally, the wave is converted back into an RF signal. The structure of SAW sensors has two main architectures [179].

- Delay Line: In this architecture, the wave can travel to a second, separate IDT for conversion back to an RF signal. Alternatively, in a reflective delay line, it is reflected back to the original IDT. By introducing multiple reflectors to create a unique pattern of reflected signals, a SAW device can also function as a time-encoded RFID tag [125]. Delay line sensors operate by measuring changes in the signal’s time delay or magnitude.
- Resonators: This design uses reflective gratings (Bragg reflectors) to trap the wave in an acoustic cavity between the reflectors. This creates a device with a very sharp and stable resonant frequency, and sensing is mainly achieved by measuring shifts in this frequency.

The detection of an external parameter is achieved when a change in this parameter affects the properties of the acoustic wave, such as velocity, resonant frequency, amplitude, and phase [177,179].

##### Applications

Surface acoustic wave (SAW) sensors are highly versatile for environmental monitoring, with significant applications in detecting airborne pollutants, water contaminants, and biological hazards [177,180]. In gas sensing, they provide rapid detection of toxic industrial chemicals and pollutants. For example, a highly sensitive sensor using graphene-based films was developed to detect nitrogen dioxide (NO2) at room temperature [181]. The sensors, based on a LiTaO3 piezoelectric substrate and a SiO2 guiding layer, achieved a sensitivity of 0.2°/ppm with an LOD of 0.068 ppm when using bilayer graphene, and 0.19°/ppm with a LOD of 0.140 ppm when using sulfur-doped graphene. For detecting carbon monoxide (CO), SAW sensors functionalized with cobalt corroles have shown remarkable performance, achieving detection limits down to 200 ppb [182]. For hydrogen (H2) monitoring, sensors using palladium–nickel (Pd/Ni) alloy films have shown excellent stability, fast response times and high sensitivity [183]. For selective gas detection, advanced sensitive layers like metal–organic frameworks (MOFs) have been used to create molecular sieves. For instance, a sensor coated with the MOF MFU-4 demonstrated a highly sensitive response to small molecules such as CO2, H2, NH3, H2O and He while completely excluding larger molecules like methane due to its ultra-narrow pore size of 2.5 Å [184]. For the detection of chemical warfare agent simulants, a passive wireless SAW sensor using a fluoroalcoholpolysiloxane (SXFA) film has been developed to detect dimethyl methylphosphonate (DMMP), a sarin simulant, with a detection limit of 0.48 mg/m^3^. The sensor operates reliably in environments ranging from −30 °C to 100 °C with humidity lower than 60% RH [185].

Conventional piezoelectric materials cannot be used in very-high-temperature environments [186]. Lithium niobate, lithium tantalate, and quartz cannot be used in environments where the operating temperature exceeds 500 °C. To address this challenge, high-temperature piezoelectric substrates such as Langasite (LGS), which can operate up to 1470 °C, and Aluminum Nitride (AlN), have been used to monitor toxic gases [187,188] and temperature [186] in harsh environments.

Passive SAW sensors can also be applied to liquid sensing. For example, pH can be monitored using coatings like ZnO nanoparticles, whose conductivity changes with pH. The sensor achieved a sensitivity of 30 kHz/pH from pH 7 to pH 2 [189].

For structural health monitoring (SHM), SAW sensors can be bonded to surfaces to measure strain or detect cracks. As a structure deforms, the induced strain alters the SAW’s frequency and propagation velocity. For example, a temperature–strain sensor fabricated on lithium niobate demonstrated a strain sensitivity of 100 Hz/μϵ at room temperature in the range of 0–700 μϵ [190]. For crack detection, a propagating crack intersecting the acoustic path causes a sudden, detectable signal attenuation [191], which has been used, for example, to monitor millimeter displacements in crack openings in concrete structures with demonstrated displacement resolution of 10 μm [192].

## 4. Remote Sensing via Drone-Mounted Sensors and AI-Assisted Decisions

Environmental remote sensing has traditionally relied on satellite-based platforms, which provide consistent, global coverage across multispectral and hyperspectral bands. Satellites such as Sentinel-2 and Landsat have been instrumental in tracking vegetation indices, urban development, and water quality over large areas. However, their coarse spatial resolution, long revisit times, and limited adaptability to localized or dynamic conditions constrain their effectiveness in fine-grained environmental studies.

**Figure 7:**
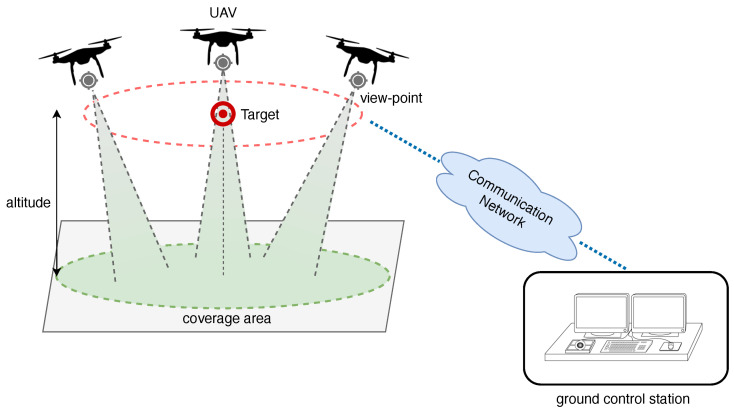
General architecture of an environmental monitoring system based on UAVs [193] under CC BY License.

To address these limitations, uncrewed aerial vehicles (UAVs) equipped with active and passive sensors are increasingly deployed for localized, high-resolution remote sensing. Drone platforms offer greater spatial precision (often <10 cm), flexible deployment schedules, and low-altitude operation, enabling fine-scale monitoring of vegetation, atmospheric conditions, and built environments. Figure 7, from Ref. [193], depicts a generic UAV-based monitoring framework, where a swarm of UAVs collects data across a designated site defined by target points. Their operations are coordinated by one or more ground control stations, which also handle data processing and preliminary analysis.

While most of the proposed solutions for environmental monitoring with UAVs are based on motorized drones, an energy-aware alternative is represented by aerostatic balloons [194,195]. These systems operate with zero direct emissions and are capable of carrying payloads of up to several kilograms, configurable according to the specific monitoring requirements. In contrast, motor-based drones (e.g., quadcopters) typically face a strict trade-off between payload and endurance, where adding even a few kilograms significantly reduces flight time due to the exponential increase in power demand from the motors, typically in the range of 20–40 min. From an operational perspective, this difference directly impacts mapping economics. In fact, covering wide areas with quadcopters requires frequent battery swaps, multiple flights, and higher logistical costs.

Drone-mounted multispectral and hyperspectral cameras are widely used in environmental monitoring to passively measure reflected sunlight and infer vegetation status, surface composition, and biophysical parameters. Vegetation indices such as NDVI or PRI can be derived from reflectance in near-infrared and red-edge bands to assess plant health, photosynthetic activity, and stress responses [196,197,198].

Active optical sensors, such as LiDAR and thermal infrared cameras, extend the sensing capabilities of UAVs by generating their own illumination. LiDAR emits laser pulses and measures the return signal to construct detailed 3D models of forest structure, canopy height, and terrain—especially valuable in densely vegetated or topographically complex areas. Lightweight UAV-compatible LiDAR systems are increasingly used for applications like biomass estimation, habitat modeling, and coastal erosion assessment [199,200,201].

Thermal sensors capture emitted infrared radiation, allowing for passive thermal imaging of surface temperatures. These have been used for precision agriculture, wildlife detection, and early fire warning systems [202,203,204]. While susceptible to atmospheric noise and solar heating effects, camera pre-calibration, correction of thermal imagery, and improved estimation of the initial image position by making use of the alignment of visual images enhance thermal data reliability for autonomous systems [205].

In addition to optical sensors, UAVs can host RF-based sensors that either passively monitor electromagnetic emissions or actively interrogate ground-based sensor nodes [206,207]. RF receivers have been used for localization of sensor tags, monitoring communication interference, and spectrum surveillance. A promising frontier involves UAVs acting as mobile energy sources, wireless powering RF-harvesting environmental sensors from above [208].

In the last years, we have witnessed an exponential growth of artificial intelligence (AI). With advancement in this field, an increasing number of environmental sensors are incorporating AI-driven detection strategies to improve classification and detection capabilities [209,210,211]. For example, Neri et al. [212] presented a sensor capable of distinguishing between different plant species and detecting plant diseases based on leaf spectral reflectance. The system utilizes a CMOS camera coupled with a Raspberry Pi for both image acquisition and onboard processing. The device, thanks to its low power consumption, can be potentially operated from environmental energy sources (e.g., solar panels).

Similarly, Falcioni et al. [213] presented an application of AI for the classification of eleven lettuce plant varieties using the full hyperspectral curves or in the ranges of 400–700 nm (VIS), 700–1300 nm (NIR), and 1300–2400 nm (SWIR). This method enables phenotyping and classification in agriculture and can be used for the detection of weeds and diseases in early stages.

Modern UAV platforms increasingly support multi-sensor payloads, combining RGB, multispectral, LiDAR, thermal, and RF sensing into a single integrated system [214,215]. Coupled with edge AI and deep learning frameworks, these enable real-time environmental classification, anomaly detection, and adaptive mission planning [214,216,217]. This synergy between sensing modalities and autonomy paves the way for scalable, energy-aware, and intelligent remote sensing platforms.

While the use of artificial intelligence on the node comes with an additional energy expenditure in the computing capabilities, it may reduce the amount of information to be transferred, lowering the overall power consumption [218].

## 5. Discussion and Outlook

In this review, we discuss recent sensing technology with related mechanisms, energy processes, and application areas, with a strong focus on energy autonomy combined with sensing functionality. A comparative analysis that includes energy requirements, environmental sustainability, readiness, reliability, and also commercialization limits is presented in Table 1 and Table 2 for active and passive sensors, respectively. Passive and active EH-assisted sensors provide significant reductions in energy needs, maintenance, and ecological footprints compared to conventional battery-sriven WSN. However, the transition from laboratory demonstrations to real commercial products requires overcoming several scientific, technological, and socio-economic barriers.

The first important challenge is reliability in the field. While some of the technologies in Table 1 (e.g., NDIR CO_2_ sensors, Raman-based systems) show high sensitivity, they suffer by stability issues and calibration needs in varying temperature, humidity, and pressure. Packaging solutions, protective coatings, and hardened power management circuits will be necessary to achieve multi-year stability for outdoor deployments.

The second barrier is scalability and reproducibility in fabrication. Table 2 passive RF and photonic crystal sensors demonstrate the possibility of sustainable monitoring using low-cost and biodegradable materials. However, large-scale production with repeatable performance is out of reach. To commercialize, compatibility with current industrial production lines and standardized test procedures will be necessary.

The issue of system-level integration poses the greatest challenge. Commercially viable platforms need to integrate not only efficient energy harvesting mechanisms but also advanced power management systems, energy storage, and adaptive communication components. Emerging developments in federated learning for distributed sensor networks [219] suggest that AI-powered data fusion can reduce communication overhead while improving autonomy and supporting predictive maintenance, as well as support for decentralized decision making.

From an environmental sustainability point of view, Table 1 and Table 2 indicate that existing technologies have a broad range of environmental impact. For instance, many active optical sensors with high performance integrate several electronic circuits, leading to inferior recyclability. On the other hand, passive RF devices using biodegradable substrates illustrate encouraging low-impact approaches. An overall life cycle analysis (LCA) is therefore necessary to balance the advantages of battery-free operation with the expenses of nanofabrication or specialized materials. The latest developments in biodegradable RFID sensors [220] illustrate the potential of bioresorbable and recyclable systems in minimizing e-waste.

Commercialization hurdles remain a major hindrance. As Table 1 and Table 2 outline, the majority of devices are at low to medium Technology Readiness Levels (TRLs 3–6). While maintenance-free operation may be cost-effective in the long term, the cost of the initial units is high, particularly for optical and nanostructured sensors. Environmental monitoring also means meeting high standards of calibration and precision for regulatory approval. Both having economic viability and certification prospects will be essential to bridging the gap between prototypes and real products.

Finally, the future for deployment looks to hybrid solutions. Hybrid devices integrating several EH sources (solar, vibrational, RF, thermal) and biodegradable substrates with AI-inspired data processing can produce self-adaptive and resilient sensing networks. Compatibility with drone-mounted or satellite-based interrogation platforms can additionally provide multi-scale monitoring. New device-level innovations, for instance, self-correcting triboelectric sensors for robust underwater salinity sensing, are representative of how field-specific challenge issues can drive commercialization. Concluding, the way forward in research has to aim not just at increased energy efficiency, multi-sensing, and adaptive integrality functions but also at closing the gaps in sustainability, reliability, and readiness for commercialization.

In addition, the coming development stage will not only require the development of material and device structures but also scalability, LCA-guided, controlled sustainability, and regulation certification processes. All these issues being resolved will enable autonomous sensors to go from laboratory prototypes to durable, cost-effective, and sustainable solutions to worldwide environmental monitoring.

## Figures and Tables

**Table 1:** Comparative analysis of active energy-autonomous sensors.

Technology	Parameters	Energy Req.	Environmental Sustainability	TRL	Maintenance	Reliability	Commercialization Barriers	References
NDIR	CO_2_ concentration	Medium (multi-circuit, not autonomous)	**Low:** high footprint (electronics, optics)	4–6	**Medium:** UAV-mounted, temperature and pressure dependance	N.A.*	High cost, UAV integration	[93]
Multi-sensors	dCO_2_, temp., humidity	10 mWh (EH modules)	**Low:** EH partly mitigates battery impact	4–6	**Low:** EH-assisted prototype	**Unknown:** prototype stage	Prototype stage, integration challenges	[94]
Light scattering	Particulate pollutants	Not autonomous	**Low:** high e-waste risk from electronics and optics	3–5	**N.A.**	**N.A.**	Low TRL, complex optics, cost	[95,97]
Raman	Molecular pollutants	High energy requirements (laser source)	**Low:** very high cost, energy intensive	3–5	**High:** potential frequent recalibration	**High:** 99.89% accuracy	High cost, complex optics	[98]
Electrochemical	Gases, pesticides, metals	Medium-high (potentiostat)	**Moderate:** electrode waste, metal disposal issues	5–7	**Medium:** electrode fouling common	**Medium–High:** proven in lab, field-dependent	Cost of nanomaterials, calibration needs	[60,76,78]
SPES	Metals, pH, pollutants	Low (biofuel/galvanic/ photo cells)	**High:** eco-friendly, biocompatible, reduced e-waste	3–5	**Low–Medium:** reduced recalibration needs	**Promising:** early validation	Low maturity, reproducibility, certification	[76,79]
TENG-based	Water quality, humidity, waves	Low–medium (µW–mW)	**High:** biodegradable options available; moderate otherwise	4–6	**Medium:** materials degrade with use	**Medium**	Durability, reproducibility	[13,34,45,47,57] and references therein
SAW-active	CO_2_ concentration	Low (piezo EH integrated)	**Moderate:** piezo materials face recycling challenges	4–6	**Medium:** substrate issues	**Medium**	Packaging, piezo material cost	[64]

* Not applicable.

**Table 2:** Comparative analysis of passive energy-autonomous sensors.

Technology	Parameters	Energy req.	Environmental sustainability	TRL	Maintenance	Reliability	Commercialization barriers	References
Photonic crystals	Humidity, temperature, analytes	None (ambient light)	**High:** biodegradable (cellulose/HPC); moderate otherwise	up to 9	**Low:** simple optical readout	**High:** proven in lab, limited field stability	Nanofabrication cost, scalability	[108,109,110,112]
Chromogenic	pH, gases, pollutants	None (ambient light)	**High:** biomass-based films, low e-waste	3–5	**Low:** recalibration sometimes needed	**Medium:** dye stability issues	Material reproducibility, dye stability	[113,114,115]
Plasmonic	Trace pollutants, gases	None (ambient light)	**Moderate:** nanoparticle recycling challenges	3–5	**Low–Medium:** some film degradation	**Medium:** NP degradation possible	NP cost, regulatory issues	[117]
Passive LC	Temperature, humidity, moisture, gases, strain, cracks	None (inductive RF)	**High:** No silicon chip, biodegradable LC substrates possible	5–9	**Low:** Recalibration and sensitive film replacement may be needed	**High:** robust sensor, sensitive readout	Read range, calibration	[124]
Chipless RFID	Temperature, humidity, moisture, gases, strain, cracks	None (ambient RF)	**High:** biodegradable tags (paper/PLA)	4–6	**Low:** Recalibration and sensitive film replacement may be needed	**Medium:** limited by range, interference	Weak signals, interference, low TRL	[121,139,141]
Chip-based RFID	Temperature, humidity, moisture, gases, strain, cracks	None (RF rectification)	**Moderate:** IC adds e-waste risk	4–9	**Low:** Recalibration and sensitive film replacement may be needed	**High:** predictable standardized performance due to IC	Reader dependence, certification	[122,125,143]
SAW (passive)	Temperature, humidity, moisture, gases, strain, cracks	None (RF backscatter)	**Moderate:** conventional substrates not recyclable	4–9	**Medium:** substrate durability limits	**High:** robust field validation	Substrate cost, harsh env. packaging	[177,179]
Living sensors	Soil conditions (bio-signature)	None (biological metabolism)	**Very High:** eco-compatible, naturally biodegradable	3–5	**Low:** self-maintaining	**Low–Medium:** still experimental	Certification, acceptance hurdles	[118]
